# T‐LAK cell‐originated protein kinase (TOPK) is a Novel Prognostic and Therapeutic Target in Chordoma

**DOI:** 10.1111/cpr.12901

**Published:** 2020-09-22

**Authors:** Pichaya Thanindratarn, Dylan C. Dean, Scott D. Nelson, Francis J. Hornicek, Zhenfeng Duan

**Affiliations:** ^1^ Department of Orthopedic Surgery Sarcoma Biology Laboratory David Geffen School of Medicine University of California Los Angeles CA USA; ^2^ Department of Orthopedic Surgery Chulabhorn hospital HRH Princess Chulabhorn College of Medical Science Chulabhorn Royal Academy Bangkok Thailand; ^3^ Department of Pathology University of California Los Angeles CA USA

**Keywords:** chordoma, immunohistochemistry, OTS514, prognostic biomarker, therapeutic target, TOPK

## Abstract

**Objectives:**

To assess the expression, prognostic value, and functionality of T‐lymphokine‐activated killer (T‐LAK) cell‐originated protein kinase (TOPK) in chordoma pathogenesis.

**Materials and Methods:**

TOPK expression in chordoma was assessed via immunohistochemical staining of a tissue microarray (TMA) and correlated with patient clinicopathology. TOPK expression in chordoma cell lines and fresh patient tissues was then evaluated by Western blot. TOPK small interfering RNA (siRNA) and the specific inhibitor OTS514 were applied to determine the roles of TOPK in chordoma pathogenicity. The effect of TOPK expression on chordoma cell clonogenicity was also investigated using clonogenic assays. A 3D cell culture model was utilized to mimic in vivo environment to validate the effect of TOPK inhibition on chordoma cells.

**Results:**

TOPK was highly expressed in 78.2% of the chordoma specimens in the TMA and all chordoma cell lines. High TOPK expression significantly correlated with metastasis, recurrence, disease status and shorter overall survival. Knockdown of TOPK with specific siRNA resulted in significantly decrease chordoma cell viability. Inhibition of TOPK with OTS514 significantly inhibited chordoma cell growth and proliferation, colony‐forming capacity and ex vivo spheroid growth.

**Conclusions:**

High expression of TOPK is an important predictor of poor prognosis in chordoma. Inhibition of TOPK resulted in significantly decrease chordoma cell proliferation and increase apoptosis. Our results indicate TOPK as a novel prognostic biomarker and therapeutic target for chordoma.

## Graphical abstract

High TOPK expression significantly correlated with metastasis, recurrence, disease status, and shorter overall survival. Inhibition of TOPK with specific inhibitor OTS514 significantly inhibited chordoma cell growth and proliferation, colony‐forming capacity, and *ex vivo* spheroid growth. These results indicate TOPK as a novel prognostic biomarker and therapeutic target for chordoma.

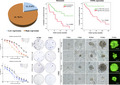

## 1 INTRODUCTION

Chordomas are rare tumours arising from remnants of the embryological notochord. The annual incidence of chordomas in the United States is approximately two cases per million people, representing 1%‐4% of all bone cancers.^1^, ^2^ The peak incidence of chordoma is between 40 and 60 years of age, with a slightly male predominance.^1^, ^3^ Chordomas most commonly arise within the sacrococcygeal area, vertebral bodies or skull base.^1^ Although chordomas have undergone histologic and genetic analysis, the molecular mechanisms driving these tumours are largely unknown. While chordomas are generally slow‐growing, they are locally invasive and aggressive tumours with notorious resistance to conventional chemotherapies and radiation.^3^, ^4^, ^5^ Currently, no effective drugs exist for chordoma treatment. Therefore, surgical resection has remained the primary treatment modality for patients; however, its insidious course and proximity to vital neurovascular structures make complete resection challenging if not possible.^2^ Additionally, some chordoma patients already have metastatic diseases upon initial diagnosis.^3^, ^6^, ^7^ The overall survival for chordoma patients is 68.4% at five years and 39.2% at 10 years, with a median overall survival of 7.8 years.^1^ The strong chemotherapeutic resistance and lack of validated prognostic biomarkers in chordoma has highlighted the need for new and robust therapeutic targets.^2^, ^3^, ^5^, ^8^

Recent studies suggest that T‐lymphokine‐activated killer (T‐LAK) cell‐originated protein kinase (TOPK) has tumorigenic roles in various malignancies.^9^, ^10^, ^11^, ^12^ TOPK, also known as PDZ‐binding kinase (PBK), is a 322‐amino acid serine/threonine kinase encoded by the PBK gene on chromosome 8p21.1. Expression and activation of TOPK function as a mitogen‐activated protein kinase kinase (MAPKK) which is essential for catalytic activity during mitosis.^9^ Recent studies have shown that TOPK regulates mitosis through its governing of several DNA binding proteins.^13^ While TOPK expression is low or undetectable in healthy tissues,^9^ it is overexpressed in lung cancer, ovarian cancer, renal cancer, colorectal cancer, prostate cancer and haematologic malignancies and correlates with worse outcomes.^11^, ^14^, ^15^, ^16^, ^17^, ^18^, ^19^ Functionally, TOPK promotes cancer cell growth and proliferation, dissemination and apoptotic resistance via numerous mechanisms.^9^ Moreover, TOPK is upregulated in and promotes the proliferation and self‐renewal of cancer stem cells, thus prompting the aggression of multiple malignancies.^20^, ^21^ These findings have given TOPK recognition as an emerging prognostic biomarker and therapeutic target with specificity for cancer cells while sparing normal host tissue. Several TOPK‐specific inhibitors have shown promising results in pre‐clinical works and are thus anticipated to be used in clinical trials in the near future.^9^, ^11^, ^12^, ^22^

In this study, we systemically investigated: (a) the expression of TOPK in chordoma patient tissues and cell lines; (b) the correlation of TOPK expression with patient clinicopathology and outcomes; (c) the function of TOPK in chordoma cell growth and proliferation; and (d) the effect of specific TOPK inhibitor on chordoma cell growth and proliferation in vitro and ex vivo three‐dimensional environment.

## 2 MATERIALS AND METHODS

### 2.1 Chordoma sample collection and tissue microarray

The tissue microarray (TMA) was constructed from 55 individual chordoma patient specimens within a formalin‐fixed paraffin‐embedded (FFPE) block as previously described.^23^, ^24^ The clinicopathological characteristics of the specimens were collected and are outlined in Table 1, including age, gender, tumour location, recurrence, metastasis and disease status. The samples included 39 (70.9%) males and 16 (29.1%) females with an average age of 58.9 years old (range: 25‐88 years old). The mean follow‐up time was 80.9 months (range: 1.4‐249.6 months). The most common tumour location was the sacrum (65.5%), followed by the lumbar spine (20.0%), thoracic spine (12.7%) and cervical spine (1.8%). Of these 55 patients, 25 (45.5%) developed disease recurrence and 12 (21.8%) developed distant metastasis.

**TABLE 1:** Correlations between TOPK expression and clinicopathology in chordoma patients

Clinicopathological features	Number of cases	TOPK expression		*P* value
	(n, %)	Low (n, %)	High (n, %)	
All patients	55 (100.0)	12 (21.8)	43 (78.2)	
Age (years)				
<60	26 (47.3)	4 (15.4)	22 (84.6)	0.283
≥60	29 (52.7)	8 (27.6)	21 (72.4)	
Gender				
Male	39 (70.9)	10 (25.6)	29 (74.4)	0.293
Female	16 (29.1)	2 (12.5)	14 (87.5)	
Tumour location				
Cervical spine	1 (1.8)	0 (0.0)	1 (100.0)	0.651
Thoracic spine	7 (12.7)	0 (0.0)	7 (100.0)	
Lumbar spine	11 (20.0)	5 (45.5)	6 (54.5)	
Sacrum	36 (65.5)	7 (19.4)	29 (80.6)	
Metastasis				
Absent	43 (78.2)	12 (27.9)	31 (72.1)	0.038*
Present	12 (21.8)	0 (0.0)	12 (100.0)	
Recurrence				
Absent	30 (54.5)	11 (36.7)	19 (63.3)	0.003*
Present	25 (45.5)	1 (4.0)	24 (96.0)	
Disease status				
No evidence of disease	19 (34.5)	8 (42.1)	11 (57.9)	0.011*
Alive with disease	12 (21.8)	3 (25.0)	9 (75.0)	
Died of disease	24 (43.7)	1 (4.2)	23 (95.8)	

* Statistical significance (*P* < .05).

### 2.2 Immunohistochemistry

The expression of TOPK was evaluated using Immunohistochemistry (IHC) assays according to the manufacturer instructions for the TOPK antibody (Cell Signaling Technology, Danvers, MA, USA). In brief, the paraffin‐embedded slide was baked for 1 hour at 60°C before xylene deparaffinization. The slide was subsequently rehydrated through graded ethanol (100% and 95%). After heated epitope retrieval, 3% hydrogen peroxide was used to quench endogenous peroxidase activity. The slide was then blocked for 1 hour with normal goat serum. Afterwards, it was incubated with a polyclonal rabbit antibody to human TOPK (1:100 dilution, Cell Signaling Technology) in a humidified chamber at 4°C overnight. SignalStain^®^ Boost Detection Reagent (Cell Signaling Technology) and SignalStain^®^ DAB (Cell Signaling Technology) were then utilized to detect the bound antibody. Finally, all the sections were counterstained with Hematoxylin QS (Vector Laboratories), and the slide was mounted with VectaMount AQ (Vector Laboratories) for long‐term preservation.

**FIGURE 1:**
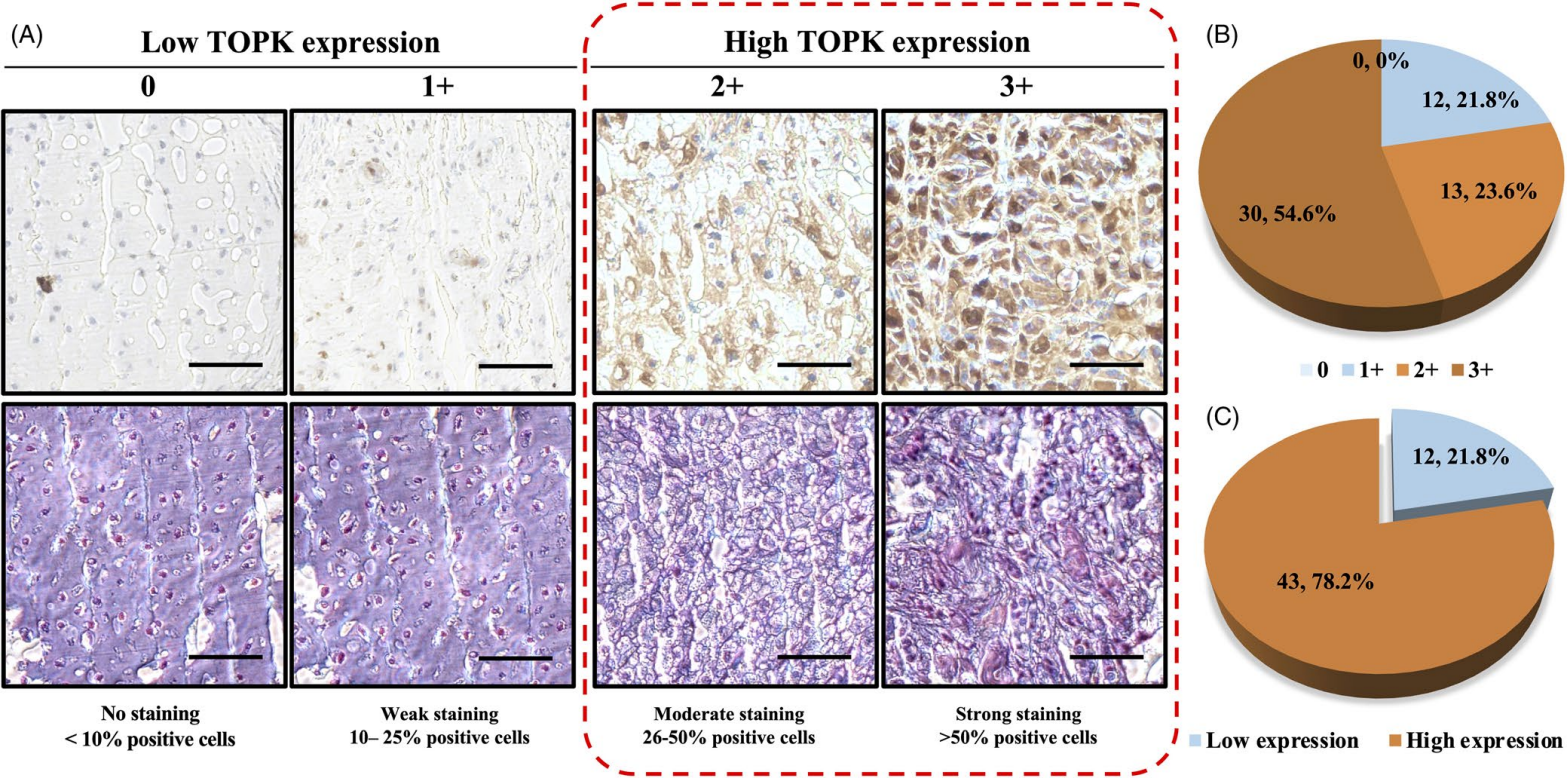
TOPK expression density by immunohistochemical staining of a chordoma TMA. (A) Representative images of different TOPK immunohistochemical staining intensities and H&E stains within chordoma tissues. Based on the TOPK staining of the chordoma samples, the cytoplasmic staining patterns were divided into 4 groups: no staining (0); weak staining (1+); moderate staining (2+); strong staining (3+). (Original magnification, 400×; Scale bar, 50 μm). (B) Pie chart of relative frequencies of different TOPK expressions in the chordoma TMAs. (C) Pie chart of relative frequencies of high TOPK expression (staining score ≥2+) and low TOPK expression (staining score ≤1+) in chordoma TMAs

The immune‐stained slides underwent microscopic evaluation (Nikon Instruments Inc). TOPK expression was subsequently categorized into four groups based on the cytoplasmic staining intensity: 0, no staining; 1+, weak staining; 2+, moderate staining; 3+, strong staining (Figure 1A). The low TOPK expression subset included groups 0 and 1+, while the groups 2+ and 3+ were defined as the high TOPK expression subset.

### 2.3 Human chordoma cell lines and culture

The human chordoma cell line UCH1 and UCH2 were established and provided by Dr Silke Brüderlein (University Hospitals of Ulm).^25^, ^26^ The CH22 chordoma cell line was established in our laboratory as previously reported.^24^, ^27^ The cell lines were cultured in Dulbecco's Modified Eagle Medium (DMEM, GIBCO, Grand Island, NY, USA). The media were supplemented with 10% foetal bovine serum (Sigma‐Aldrich) and 2% penicillin/streptomycin (Life Technologies). All cell lines were incubated in a humidified 5% CO_2_ atmosphere at 37°C.

### 2.4 Protein preparation and western blotting

The protein was extracted from the cells and chordoma tissue specimens with 1x RIPA lysis buffer (Sigma‐Aldrich) supplemented with protease inhibitor cocktail tablets (Roche Applied Science). The protein lysate concentrations were then determined by DC^TM^ protein assay reagents (BIO‐RAD, Hercules, CA, USA) and a spectrophotometer SPECTRA max 340PC (Molecular Devices, LLc.). Western blotting was performed using a similar method to those previously described. In brief, equal amounts of protein were separated on 4%‐12% Bis‐Tris gels (NuPAGE^®^, Thermo Fisher Scientific) before they were transferred to nitrocellulose membranes. The membranes were incubated with the following specific primary antibodies at 4°C overnight after they were blocked in 5% non‐fat milk for 1 hour, TOPK (1:500 dilution, Cell Signaling Technology), poly (ADP‐ribose) polymerase (PARP) (1:1000 dilution, Cell Signaling Technology), Mcl‐1 (1:1000 dilution, Santa Cruz Biotechnology), Survivin (1:1000 dilution, Cell Signaling Technology) and β‐actin (1:1000 dilution, Sigma‐Aldrich). Following incubation with the primary antibodies, the membranes were washed with TBST three separate times for five minutes and then further incubated with Goat anti‐Rabbit IRDye^®^ 800CW (926‐32 211, 1:10 000 dilution) and Goat anti‐Mouse IRDye^®^ 680LT secondary antibody (926‐68 020, 1:10 000 dilution) (Li‐COR Biosciences) for 1 hour at room temperature. After being washed with TBST another three times, the bands were detected using Odyssey^®^ CLx equipment (LI‐COR Bioscience) and Odyssey software 3.0. The quantity of β‐actin was measured to ensure equal loading.

### 2.5 Immunofluorescence assay

The expression of TOPK in chordoma cells was visualized by immunofluorescence assays. The UCH2 and CH22 cells were grown for three days in 24‐well plates and fixed with 4% paraformaldehyde for 15 minutes before being permeabilized with ice‐cold 100% methanol and blocked with 1% bovine serum albumin. Immunostaining was performed with TOPK (1:200 dilution, Cell Signaling Technology) and β‐actin (1:500 dilution, Sigma‐Aldrich) antibody at 4°C overnight. The next day, the cells were incubated for an additional 1 hour with Alexa Fluor 488 (Green) conjugated goat anti‐rabbit antibody or Alexa Fluor 594 (red) goat anti‐mouse antibody (Invitrogen). Nuclei were counterstained with 1 μg/mL Hoechst 33 342 (Invitrogen). Cell images were obtained using a Nikon Eclipse Ti‐U fluorescence microscope (Diagnostic Instruments Inc) equipped with a SPOT RT^TM^ digital camera. Green colour highlights TOPK protein, blue highlights nucleus, and red highlights cytoplasm.

### 2.6 Knockdown of TOPK by siRNA transfection and MTT assay

Knockdown of TOPK in chordoma cells was performed via specific small interfering RNA (siRNA) transfection. In brief, UCH2 and CH22 cells were grown at a density of 4 × 10^3^ cells/well in 96‐well plates or 4 × 10^4^ cells/well in 12‐well plates and transfected with increasing concentrations (0, 10, 30 or 60 nmol/L) of synthesized TOPK siRNA (5ʹ‐GACCAUAGUUUCUUGUUAA‐3ʹ) (Sigma‐Aldrich) using the Lipofectamine RNAiMax reagent (Invitrogen) according to manufacturer instructions. Non‐specific siRNA (SIC001; Sigma‐Aldrich) was used as a negative control. Three days following transfection with TOPK siRNA, the proteins of UCH2 and CH22 cells were extracted for measurement via Western blot. Cellular proliferation was assessed by conventional 3‐(4,5‐dimethylthiazol‐2‐yl)‐2,5‐diphenyl tetrazolium bromide (MTT) assays. At the end of the 5‐day treatment, 20 μL of MTT (5 mg/mL; Sigma‐Aldrich) was added to each well of the 96‐well plates. After incubating at 37°C in a humidified 5% CO_2_ atmosphere for 4 hours, the resulting formazan product was solubilized with 100 μL of acid isopropanol and the absorbance was measured at a wavelength of 490 nm on the SpectraMax Microplate^®^ Spectrophotometer (Molecular Devices LLC).

### 2.7 Suppression of TOPK by OTS514 treatment and MTT assay

The highly selective and potent TOPK inhibitor OTS514, ((R)‐9‐(4‐(1‐aminopropan‐2‐yl)phenyl)‐8‐hydroxy‐6‐methylthieno(2,3‐c)quinoline‐4(5H)‐one, (Selleckchem), has been shown to inhibit the effect of TOPK in lung cancer, ovarian cancer, kidney cancer and haematologic malignancies both in vitro and in vivo.^11^, ^16^, ^17^, ^18^, ^19^ OTS514 inhibited TOPK kinase activity at a half‐maximal inhibitory concentration (IC50) value of 2.6 nmol/L.^20^ More recently, fluorescently‐labelled OTS514 has been used to generate intraoperative in vivo tumour imaging.^28^ Here, UCH2 and CH22 cells were seeded into 96‐well plates at a density of 4x10^3^ cells/well or 4 × 10^4^ cells/well in 12‐well plates and incubated with increasing concentrations (0, 1, 2, 5 and 10 nmol/L) of OTS514 for 2, 3 or 5 days prior to the following experiments. Three days following treatment with OTS514, the proteins of UCH2 and CH22 cells were extracted for protein measurement via Western blot. After OTS514 treatment for 5 days, the cell proliferation of UCH2 and CH22 was investigated using the MTT assay (as previously mentioned in the experimental protocol above). A Nikon microscope (Nikon Instruments Inc) was used to evaluate the morphological changes of UCH2 and CH22 cells after 3 and 5 days of OTS514 treatment.

### 2.8 Clonogenic assay

The clonogenic assay, as known as the colony formation assay, is a well‐established in vitro cell survival experiment based on the ability of a single cancer cell to grow into a colony.^29^ A clonogenic assay can be used to study the effect of a specific agent on cancer cell proliferation and survival. Chordoma UCH2 and CH22 cells were seeded at 200 cells/well in the 12‐well plates and treated with the TOPK inhibitor, OTS514, at different concentrations (0, 2.5, 5, 10 nmol/L), and then incubated at 37°C for 10 days. Colonies were subsequently fixed with methanol for 10 minutes and then washed with PBS three times before staining with 10% Giemsa stain (Sigma‐Aldrich) for 20 minutes. The cells were washed with flowing water and allowed to dry. Pictures of the stained colonies were obtained using a digital camera (Olympus).

### 2.9 Three‐dimensional cell culture

Three‐dimensional (3D) cell culture is an artificially created environment that allows cells in vitro to interact with their surroundings and to grow in all directions, similar to in vivo growth.^30^ The hydrogel 3D culture system (VitroGel 3D‐RGD, #TWG002, TheWell Bioscience) was prepared according to the manufacturer's protocol. In short, 250 μL of a 2 × 10^4^ cells/mL suspension of UCH2 or CH22 was mixed with the prepared hydrogel 3D culture system and seeded into 24‐well culture plates. An additional 250 μL of DMEM supplemented with 10% FBS and 2% penicillin/streptomycin was added to cover the hydrogel. Immediately following this, 5 nmol/L of OTS514 was added into the medium. Spheroids formed from untreated chordoma cells served as the negative control. The culture plates were then incubated at 37°C in a humidified 5% CO_2_ atmosphere. The medium was changed every five days to provide sufficient nutrients and to prevent an osmolarity shift in the culture system. Pictures of chordoma spheroids were captured under a microscope (Nikon Instruments Inc) with NIS‐Elements platform every other day. At ten days, the spheroids were also imaged on a Nikon Eclipse Ti‐U inverted fluorescence microscope (Nikon Instruments Inc) after 15 minutes of incubation with 0.25 μmol/L Calcein AM (Life Technologies).

### 2.10 Statistical analysis

GraphPad Prism 8 software (GraphPad Software) and SPSS 23.0 (IBM Corp.) were used for statistical analyses. Non‐parametric testing (Mann‐Whitney U test) was performed to compare two independent groups and determine statistical significance. A one‐way analysis of variance (ANOVA) was performed for multiple comparisons. The survival curves were produced by Kaplan‐Meier methods. The relationship between different clinical parameters and overall survival (OS) was evaluated by Cox regression analysis. Only those factors that were statistically significant (*P* < .05) in the univariate analysis were included in the multivariate analysis. The median OS and hazard ratio (HR) were reported along with a 95% confident interval (CI). A *P* < 0.05 was considered statistically significant.

## 3 RESULTS

### 3.1 TOPK is highly expressed in chordoma cell lines, fresh tissues and a constructed TMA

We first validated TOPK expression within a constructed human chordoma TMA. All 55 (100.0%) of the patient tissues showed positive TOPK immunostaining in the cytoplasm, ranging from 1+ staining (12 of 55, 21.8%); 2+ staining (13 of 55, 23.6%) to 3+ staining (30 of 55, 54.6%) (Figure 1A,B). These stained specimens were then subdivided into two categories: 0, 1 + were defined as being low TOPK expression (21.8%) and 2+, 3+ as high TOPK expression (78.2%) (Figure 1C, Table 1).

**FIGURE 2:**
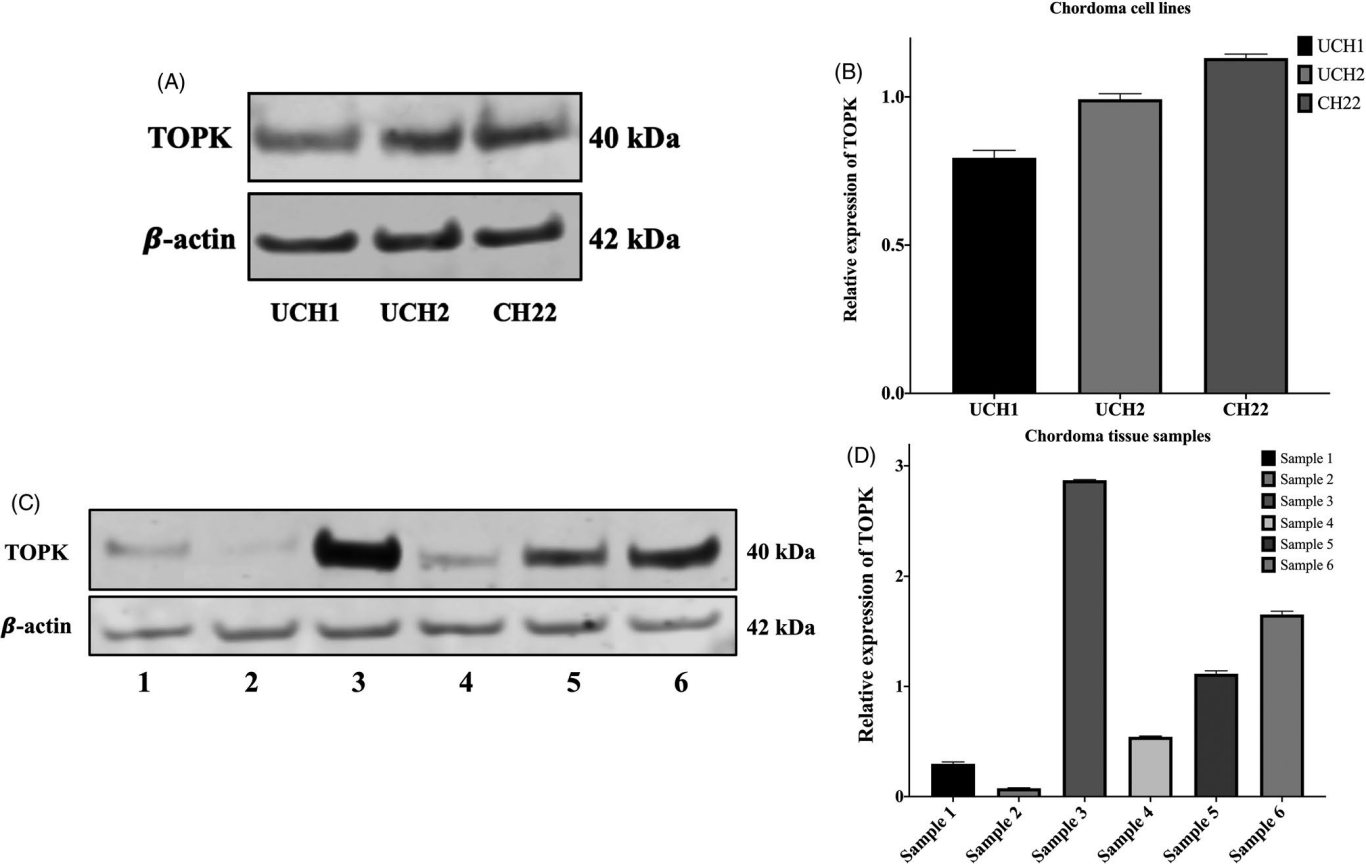
TOPK gene and protein expression in chordoma tissues and cell lines. (A) Differential expression of TOPK in various chordoma cell lines via Western blot. (B) Densitometry quantification of Western blot results from (A), presented as relative to β‐actin expression. The data represent the mean ± SD of the experiment carried out in triplicate. (C) Expression of TOPK in chordoma tissue samples from six patients via Western blot. (D) Densitometry quantification of Western blot results from (C), presented as relative to β‐actin expression. The data represent the mean ± SD of the experiment carried out in triplicate

**FIGURE 3:**
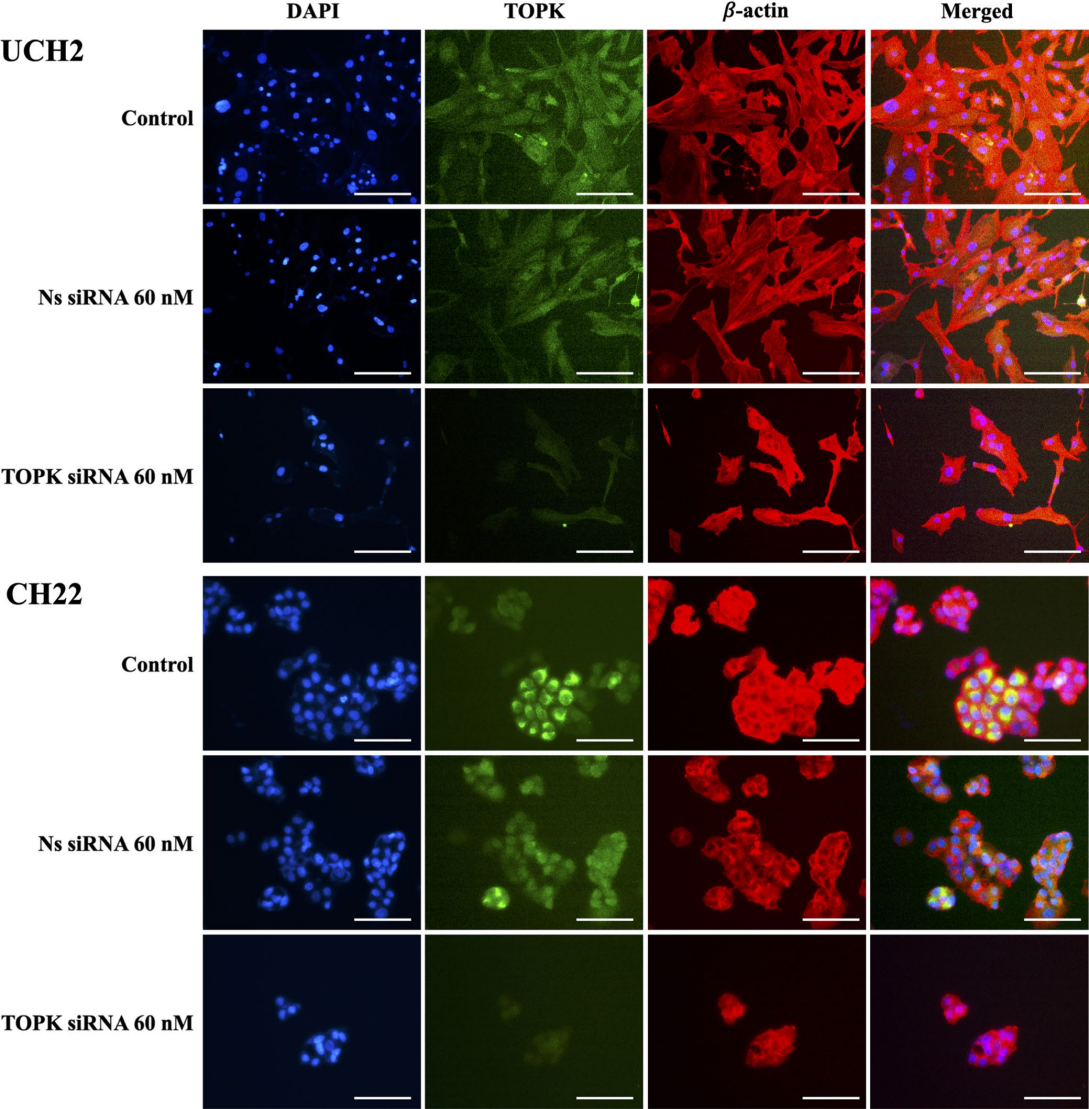
TOPK expression in chordoma cell lines determined by immunofluorescence. TOPK expression in chordoma cell lines, with groups including cell only, transfection with non‐specific siRNA (60nM) and TOPK siRNA (60 nmol/L). Immunofluorescence signals include TOPK (green), β‐actin (red in cytoplasm) and Hoechst 33342 (blue in nuclei). The green fluorescence signal indicating TOPK protein was localized in the cytoplasm of chordoma cells and was clearly inhibited by TOPK siRNA. (Scale bar; 50 μm)

We also found TOPK overexpression in the chordoma cell lines UCH1, UCH2 and CH22 via Western blot analysis (Figure 2A,B). In order to exclude the possibility that TOPK expression is an artefact of in vitro propagation, we also investigated the expression of TOPK in six fresh human chordoma specimens and found varying degrees of positive expression in all tested specimens (Figure 2C,D). Interestingly, the chordoma tissues from patients with either recurrent or metastatic disease showed higher TOPK expression (Table 2). Among the high TOPK expressing chordoma tissues, two of the patients were alive with disease. In contrast, no evidence of disease was observed in the patients who had low TOPK expressing chordomas (Table 2). We also examined the localization of TOPK via immunofluorescence of UCH2 and CH22 cell lines and found that the TOPK protein was located primarily within the cytoplasm (Figure 3). This cell line work was consistent with the chordoma TMA, which also demonstrated high TOPK expression within chordoma tissues and a cytoplasmic localization.

**TABLE 2:** Clinicopathologic characteristics of fresh chordoma tissues

Sample	Age (years)	Gender	Pathology	Location	Margin	Recurrence	Metastasis	Disease status
1	33	Male	Chordoma	Cervical spine	Negative	No	No	NED
2	29	Male	Chordoma	Sacrum	Negative	No	No	NED
3	73	Male	Chordoma	Sacrum	Negative	Yes	Yes	AWD
4	64	Female	Chordoma	Thoracic spine	N/A	No	No	NED
5	82	Female	Chordoma	Clivus	N/A	Yes	No	AWD
6	64	Female	Chordoma	Sacrum	Negative	Yes	No	NED

Abbreviations: AWD, alive with disease; NED, no evidence of disease; N/A, not available.

### 3.2 TOPK expression in chordoma correlates with patient clinical characteristics and prognosis

**FIGURE 4:**
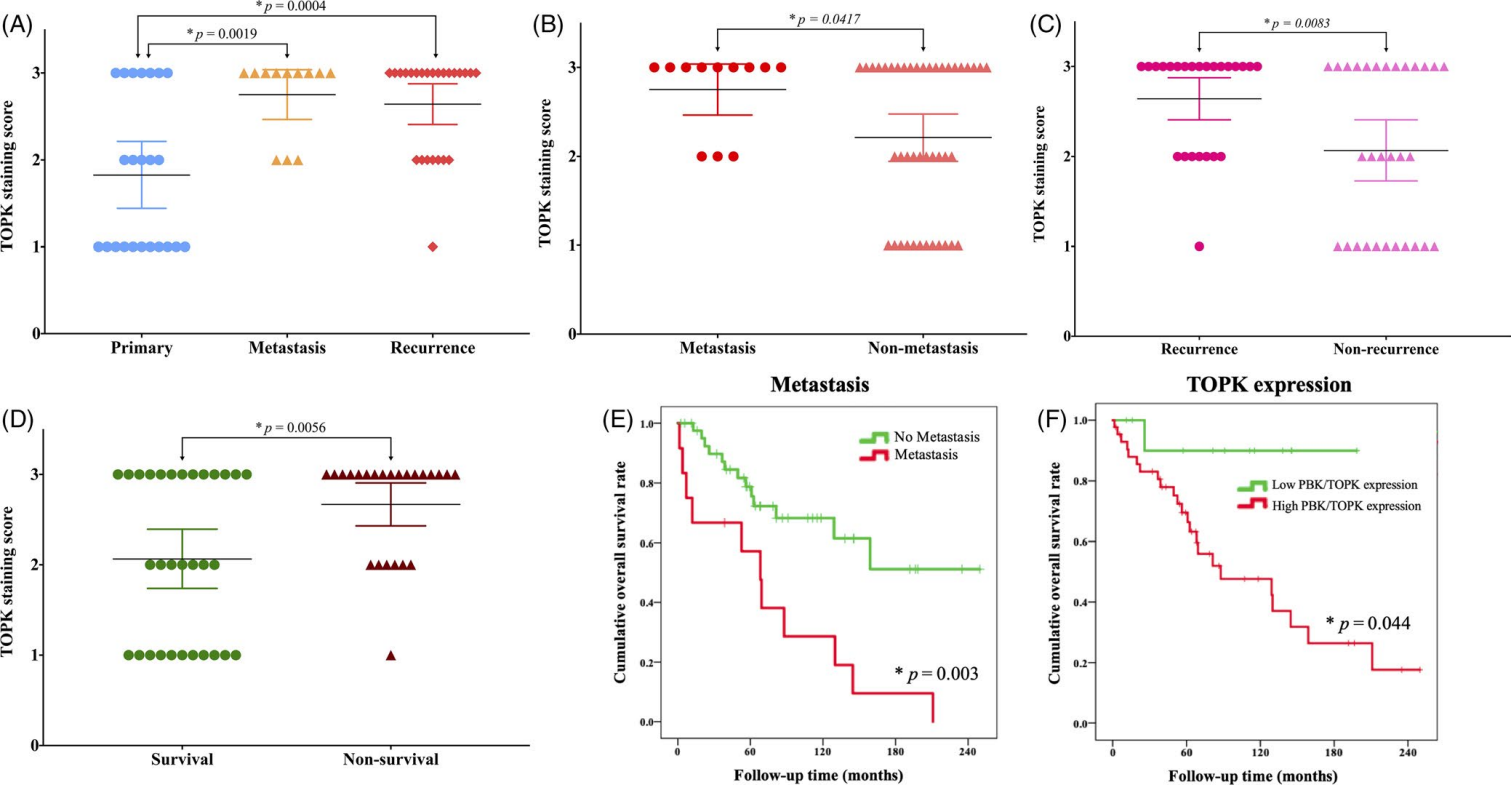
Correlation between TOPK expression and chordoma patient clinicopathology and prognosis. (A) Distribution of TOPK immunostaining scores in primary chordoma tissues compared to tissues from patients with recurrent and metastatic disease. (B) Comparison of TOPK immunohistochemistry staining scores between chordoma tissues from patients with metastatic and non‐metastatic disease (based on disease status of patients at the end of follow‐up time). (C) Comparison of TOPK immunohistochemistry staining scores between chordoma tissues of patients with recurrent and non‐recurrent diseases (based on disease status of patients at the end of follow‐up time). (D) Comparison of TOPK immunohistochemistry staining scores between chordoma tissues from survival and non‐survival patients based on disease status at the end of follow‐up time. (E) Kaplan‐Meier overall survival curve of chordoma patients in our study was sub‐grouped into either the metastatic or non‐metastatic group. Compared to the non‐metastatic group, patients with metastasis had significantly shorter overall survival. (F) Kaplan‐Meier overall survival curve of chordoma patients sub‐grouped as having either low TOPK expression (staining score ≤1+) or high expression (staining score ≥2+). Compared to the low expression group, patients with high TOPK expression had significantly shorter overall survival. *P < .05

After confirming the high expression of TOPK in the chordoma TMA, we performed follow‐up statistical analysis for clinical significance. We found higher TOPK expression significantly correlated with recurrent or metastatic chordoma compared to primary disease alone (*P* = .0004 and *P* = .019, respectively) (Figure 4A). TOPK expression was also significantly higher in the chordoma tissues from patients who developed metastatic disease compared with those who did not (*P* = .0417) (Figure 4B). In addition, TOPK was significantly overexpressed in the chordoma tissues from patients with recurrent disease compared to those without recurrence (*P* = .0083) (Figure 4C). Moreover, the chordoma tissues from patients who died demonstrated significantly higher TOPK expression than those patients who survived (*P* = .0056) (Figure 4D). Further analysis showed expression of TOPK had a statistically significant correlation with metastasis, recurrence and disease status in chordoma (*P* = .038, *P* = .003 and *P* = .011, respectively), while other clinical parameters such as patient age, gender and tumour location had no such significance (Table 1).

Next, an overall survival analysis was performed to evaluate the prognostic predictive value of TOPK expression in chordoma. In our TMA analysis, the OS was 80.51% at three years, 63.27% at five years, and 48.42% at ten years. The median OS was 129.93 months (59.29‐200.57). The OS of patients with high TOPK expressing chordomas was 77.86% at three years, 55.57% at five years and 37.02% at ten years, with a median OS at 89.05 months. In contrast, the OS of patients with low TOPK expressing chordomas was 90.00% at three, five and ten years, with a median OS of 192.00 months (Table 3). A univariate analysis demonstrated metastasis (HR = 3.40 (1.52‐7.62), *P* = .003) and TOPK expression (HR = 7.90 (1.06‐58.78), *P* = .044) were poor prognostic factors for OS (Table 3) (Figure 4E,F). However, our multivariate analysis showed only metastasis was an independent risk factor for OS in patients with chordoma.

**TABLE 3:** Univariate and multivariate overall survival analysis of prognostic factors in chordoma

Variable	Overall survival (%)			Median overall survival (months)	Univariate analysis		Multivariate analysis	
	3‐year	5‐year	10‐year		HR (95% CI)	*P* value	HR (95% CI)	*P* value
Age (years)								
<60	84.14	78.53	53.68	160.11	1.49 (0.66‐3.37)	0.342		
≥60	77.16	50.01	42.86	120.01				
Gender								
Male	83.12	64.88	58.39	204.43	0.47 (0.21‐1.08)	0.076		
Female	73.70	58.83	26.55	83.71				
Tumour site								
Cervical spine	100.00	100.00	‐	78.00	0.97 (0.55‐1.71)	0.925		
Thoracic spine	83.33	59.52	‐	72.00				
Lumbar spine	77.54	64.62	64.62	192.00				
Sacrum	80.04	62.48	46.54	128.43				
Recurrence								
Absent	78.79	65.59	65.59	155.41	1.49 (0.66‐3.39)	0.339		
Present	82.50	60.16	33.42	90.23				
Metastasis								
Absent	84.36	71.37	60.67	240.00	3.40 (1.52‐7.62)	0.003*	2.51 (1.10‐5.73)	0.028*
Present	66.67	38.10	19.05	64.50				
TOPK								
Low expression	90.00	90.00	90.00	192.00	7.90 (1.06‐58.78)	0.044*	5.58 (0.72‐43.14)	0.100
High expression	77.86	55.57	37.02	89.05				

Abbreviations: CI, confident interval; HR, hazard ratio.

* Statistical significance (*P*<0.05).

### 3.3 TOPK knockdown by siRNA decreased proliferation of human chordoma cell lines

**FIGURE 5:**
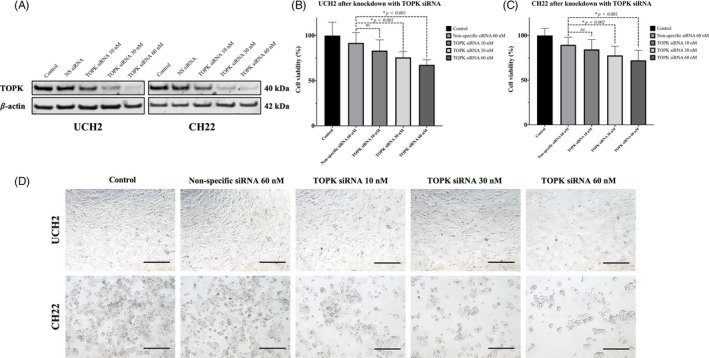
Effects of TOPK knockdown with specific siRNA on chordoma cell line growth. (A) Expression of TOPK in UCH2 and CH22 chordoma cells after transfection with different concentrations of TOPK‐specific siRNA via Western blot. (B) Cell viability of UCH2 after transfection with increasing concentrations of TOPK‐specific siRNA. The data represent the mean ± SD of the experiment carried out in triplicate. (C) Cell viability of CH22 after transfection with increasing concentrations of TOPK‐specific siRNA. The data represent the mean ± SD of the experiment carried out in triplicate. (D) Microscopic images exhibit cell number reduction after TOPK knockdown with specific siRNA for five days in both KHOS and U2OS cell lines. (Scale bar; 100 μm). * *P* < .05, ns; no statistical significance

To evaluate the role of TOPK in chordoma cell proliferation and growth, we knocked down TOPK expression with TOPK siRNA and observed changes. We used an immunofluorescence assay and Western blots to assess the expression of TOPK in chordoma cell lines following TOPK siRNA transfection. Immunofluorescence studies revealed a marked reduction in TOPK in both UCH2 and CH22 following 60 nmol/L of TOPK siRNA transfection (Figure 3). Western blots further confirmed a significant reduction of TOPK expression with increasing concentrations of siRNA transfection in both UCH2 and CH22 compared to the cell only or non‐specific siRNA transfected groups (Figure 5A).

In MTT assays, a dose‐dependent decrease in cell viability was observed in both UCH2 and CH22 cells with transfection of increasing concentrations of TOPK siRNA over five days. Similar findings were not observed in the control groups, including the non‐specific siRNA transfected cells and the untreated cells (Figure 5B‐D).

### 3.4 Pharmacological TOPK inhibition with OTS514 in chordoma cell lines

**FIGURE 6:**
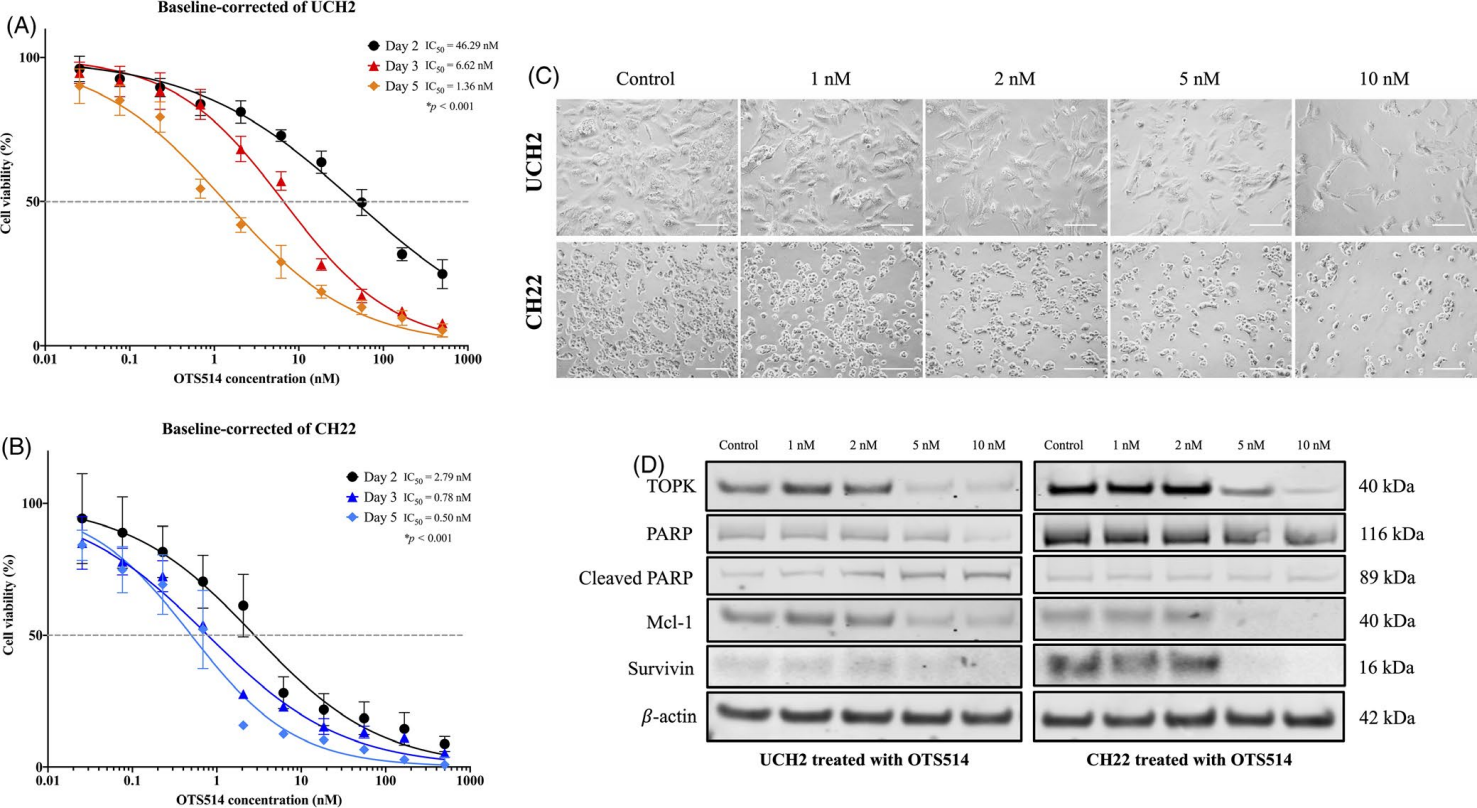
Effects of TOPK inhibition by OTS514 on chordoma cell lines. (A) Dose‐response curve of UCH2 treated by OTS514 for two, three and five days. The data represent mean ± SD of the independent triple experiment. (B) Dose‐response curve of CH22 treated by OTS514 for two, three and five days. The data represent mean ± SD of the independent triple experiment. (C) Microscopic images exhibit cell number reduction after TOPK inhibition with OTS514 for five days in both UCH2 and CH22 cell lines. (Scale bar; 100 μm). (D) Expression of proteins involved in the anti‐apoptotic activity of TOPK in UCH2 and CH22 chordoma cells after treated with OTS514 for three days, as examined by Western blot. *P < .001

After validating the expression and clinical significance of TOPK in chordoma tissue samples, we further assessed the effects of TOPK inhibition on the proliferation of UCH2 and CH22 chordoma cells with the TOPK inhibitor OTS514. Cell viability was decreased in a dose‐ and time‐dependent manner in UCH2 and CH22 chordoma cell lines, with IC50 values for five‐day OTS514 treatment at 1.36‐46.29 and 0.50‐2.79 nmol/L, respectively (Figure 6A,B). Similarly, morphological changes and reduced cell viability were observed with increasing concentrations of OTS514 in UCH2 and CH22 cells after 72 hours (Figure 6C). Following incubation of the UCH2 and CH22 cell lines with 1, 2, 5 and 10 nmol/L of OTS514 for 72 hours, Western blots showed TOPK and the anti‐apoptotic protein Mcl‐1 and Survivin significantly decreased while apoptotic cleavage of PARP increased in a dose‐dependent manner (Figure 6D).

**FIGURE 7:**
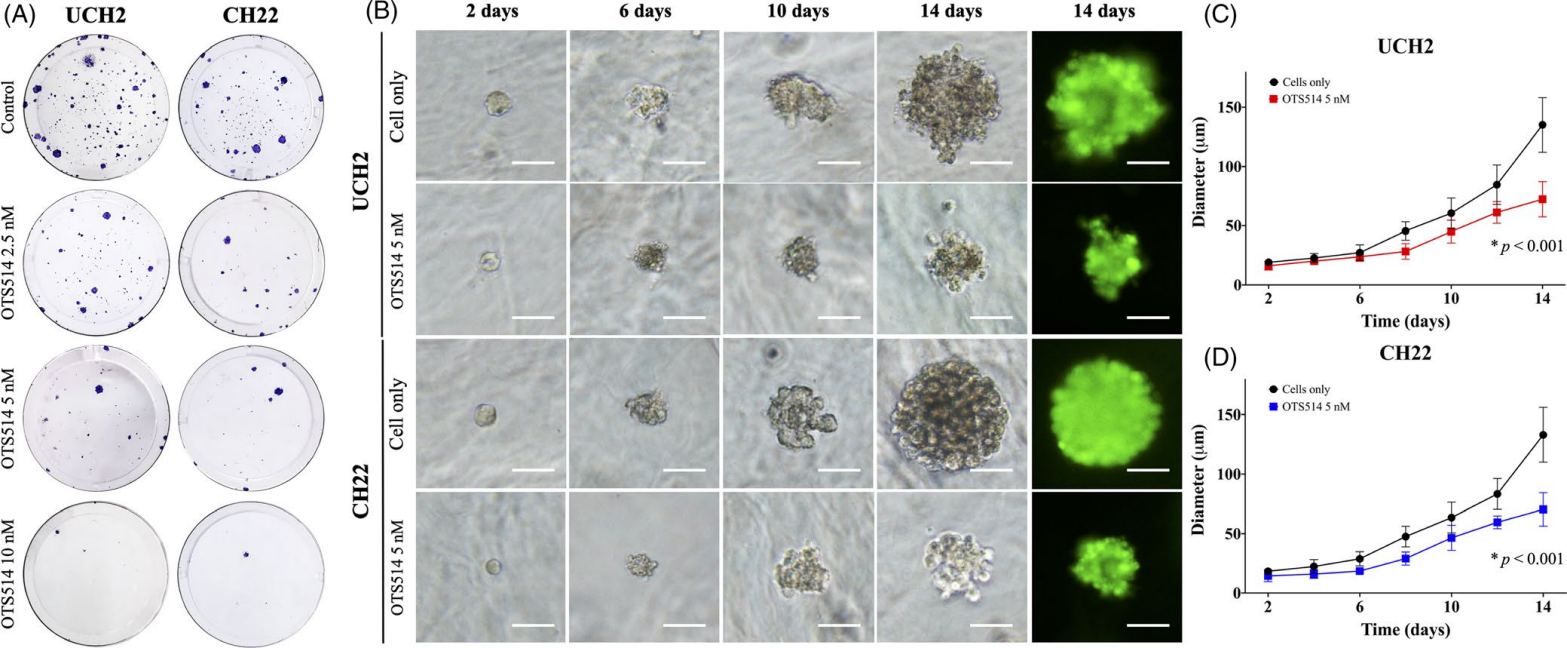
TOPK inhibition by OTS514 reduced chordoma cell clonogenicity and spheroid diameter of cells in a 3D cell culture. (A) Representative images of colony formation in UCH2 and CH22. The number of colonies and their sizes were markedly decreased in chordoma cells treated with OTS514. (B) Representative images of spheroid formation of UCH2 and CH22 chordoma cells cultured with 5 nmol/L of OTS514. Fluorescence images of spheroid formation were captured after 14 days of culturing. Spheroid formation of UCH2 and CH22 cultured with OTS514 was significantly smaller than untreated cells at all observation points. (Scale bar; 50 μm). (C) The curve of spheroid diameters of UCH2 cells treated with OTS514 showed significant decrease compared with untreated cells. The data represent mean ± SD of the independent triple experiment. (D) The curve of spheroid diameters of CH22 cells treated with OTS514 showed significant decrease compared with untreated cells. The data represent mean ± SD of the independent triple experiment. *P < .001

We next assessed the effect of OTS514 on the colony‐forming ability of chordoma cells with a clonogenic assay. The clonogenicity of UCH2 and CH22 was reduced in a dose‐dependent manner when treated with increasing concentrations of OTS514 compared to the untreated cells (Figure 7A). In addition, we evaluated whether TOPK suppression via OTS514 would alter the tumorigenicity of chordoma cells in a simulated in vivo environment of 3D cell culture. The spheroid diameters of the OTS514‐treated UCH2 and CH22 cells were significantly smaller than the untreated cells (Figure 7B‐D). After 14 days of 10 nmol/L of OTS514 treatment, the spheroid diameter of UCH2 cells was 53.5% of the untreated UCH2 cells (*P* < .001, Figure 7C). Similar results were also seen in the CH22 cell line, with the spheroid diameter of the CH22 cells being 52.8% of the untreated CH22 cells after the treatment period (*P* < .001, Figure 7D).

## 4 DISCUSSION

TOPK has become an attractive therapeutic target due to its oncogenic roles and comparatively high expression in malignancies to their normal tissue counterparts.^11^, ^14^, ^15^, ^16^, ^17^ Therefore, we evaluated TOPK expression in chordoma tissues and cell lines to discern whether it was also significant in this rarer and untested neoplasm. Our TMA revealed all chordoma tissues expressed TOPK, of which 78.2% had a high expression. Similarly, elevated TOPK protein expression occurred in half of our fresh chordoma tissues, with the tissues of patients with disease recurrence or metastasis having especially high TOPK expression. High TOPK expression was also confirmed in all of our tested human chordoma cell lines.

While the complete roles of TOPK in chordoma are unknown, several studies have shown that TOPK is a prognostic marker of poor outcomes in lung cancer, ovarian cancer, kidney cancer, colorectal cancer, leukaemia, melanoma and glioblastoma.^11^, ^14^, ^15^, ^16^, ^17^, ^18^, ^19^, ^31^ Although brachyury is a vital biomarker for notochord‐derived tissues such as chordoma, it is not a prognostic indicator in clinical use.^8^, ^32^, ^33^ In this study, we found high TOPK expression significantly correlated with recurrence, metastasis and shorter overall survival. Specifically, all 12 tissues from patients with metastasis and 24 of 25 (96.0%) tissues from patients with recurrence had high TOPK expression. In addition, chordoma patients with high TOPK expression had a comparatively shorter overall survival rate, with a hazard ratio of 7.90 by univariate analysis. Taken together, these results support the prognostic significance of TOPK expression for chordoma patients.

TOPK knockdown via siRNA or short hairpin RNA (shRNA) has been shown to decrease tumour cell growth and induce apoptosis in various cancers.^10^, ^34^, ^35^, ^36^, ^37^ To explore the roles of TOPK in chordoma, we performed a knockdown analysis using TOPK‐specific siRNA. We found a significant reduction in cell growth and viability in both UCH2 and CH22 chordoma cell lines.

Several compounds have been developed to specifically inhibit TOPK, such as OTS514, HI‐TOPK‐032 and ADA‐07, with OTS514 showing the highest potency and specificity.^9^, ^11^, ^18^ Pre‐clinical and xenograft studies have shown that OTS514 effectively inhibits tumour cell growth and dissemination in small cell lung cancer, ovarian cancer, kidney cancer and haematologic malignancies.^11^, ^16^, ^17^, ^18^, ^19^ We performed in vitro TOPK loss‐of‐function studies to assess its roles in chordoma cell pathogenesis. Similar to our siRNA‐mediated TOPK knockdown findings, the inhibition of TOPK with OTS514 decreased UCH2 and CH22 chordoma cell growth and proliferation in a dose‐ and time‐dependent manner. While the precise molecular effects of TOPK inhibition on chordoma are undefined, we show a significant reduction of the anti‐apoptotic proteins Mcl‐1 and Survivin and increased apoptotic cleavage of PARP. These results show TOPK has roles in cell proliferation while blocking apoptosis in chordoma. Previous studies have found OTS514 has anticancer effects in TOPK expressing tumours by regulating FOXM1 and MELK expression.^11^, ^16^, ^17^, ^18^ More recently, TOPK has been shown to positively regulate the TBX3 in TGF‐β/Smad signalling pathway in breast cancer, thereby enhancing epithelial‐mesenchymal transition (EMT) and tumour cell invasion.^36^ Interestingly, the TGF‐β signalling pathway acts upstream of brachyury and has vital roles in bone and cartilage development.^38^, ^39^ Overexpression and activation of either the TGF‐β or Smad signalling pathways have been found in chordomas and can predict poor clinical outcomes as well.^40^, ^41^ However, correlations and detail underlying mechanisms of TOPK with these signalling pathways in chordoma need to be investigated.

Clonogenic assays are in vitro cell survival experiments that function based on the premise that single cancer cells can quickly proliferate and form colonies.^42^, ^43^ We found the number and size of UCH2 and CH22 chordoma cell colonies reduced in a dose‐dependent manner following OTS514 treatment (Figure 7A). Next, as 3D cell culture models are validated in vitro models mimicking the in vivo environment,^30^, ^44^ we investigated the effects of OTS514 on chordoma cells in this unique tumour culturing system. We found the spheroid diameters of chordoma cells treated with OTS514 were significantly decreased compared with untreated cells (*P* < .001, Figure 7B‐D). Previous studies have also shown in vivo suppression of tumour growth and proliferation in murine xenografts following treatment with OTS514.^11^ Taken together, TOPK is an emerging prognostic biomarker and therapeutic target for chordoma patient treatment.

## 5 CONCLUSION

Our study demonstrates TOPK is highly expressed in chordoma and significantly correlates with recurrence, metastasis and shorter overall survival. Inhibition of TOPK with siRNA or inhibitor significantly reduces chordoma cell growth and proliferation. Our findings support TOPK as a potential prognostic biomarker and therapeutic target in chordoma therapy, warranting future mechanistic and in vivo investigations.

## CONFLICT OF INTEREST

The authors declare that there is no conflict of interest.

## AUTHOR CONTRIBUTIONS

Pichaya Thanindratarn contributed to conception, design, experiment, data acquisition, analysis and interpretation, drafted and critically revised the manuscript. Dylan C. Dean, Scott D. Nelson, and Francis J. Hornicek contributed to analysis and interpretation and critically revised the manuscript. Zhenfeng Duan contributed to conception, design, analysis and interpretation and critically revised the manuscript. All authors gave final approval and agreed to be accountable for all aspects of the present work.

## ETHICAL APPROVAL AND CONSENT TO PARTICIPATE

All experiments were reviewed and approved by the Ethics Committee of David Geffen School of Medicine, University of California, Los Angeles.

## Data Availability

The data that support the findings of this study are available from the corresponding author upon reasonable request.
